# New Findings in eNOS gene and Thalidomide Embryopathy Suggest pre-transcriptional effect variants as susceptibility factors

**DOI:** 10.1038/srep23404

**Published:** 2016-03-23

**Authors:** Thayne Woycinck Kowalski, Lucas Rosa Fraga, Luciana Tovo-Rodrigues, Maria Teresa Vieira Sanseverino, Mara Helena Hutz, Lavínia Schuler-Faccini, Fernanda Sales Luiz Vianna

**Affiliations:** 1 INAGEMP, National Institute of Medical Population Genetics, Porto Alegre, Brazil; 2 Post-graduate program in Genetics and Molecular Biology, Universidade Federal do Rio Grande do Sul, Porto Alegre, Brazil; 3 Post-graduate program in Epidemiology, Universidade Federal de Pelotas, Pelotas, Rio Grande do Sul, Brazil; 4 Teratogen Information Service, Medical Genetics Service, Hospital de Clínicas de Porto Alegre, Brazil; 5 Post-Graduate program in Epidemiology, Universidade Federal do Rio Grande do Sul, Porto Alegre, Rio Grande do Sul, Brazil

## Abstract

Antiangiogenic properties of thalidomide have created an interest in the use of the drug in treatment of cancer. However, thalidomide is responsible for thalidomide embryopathy (TE). A lack of knowledge regarding the mechanisms of thalidomide teratogenesis acts as a barrier in the aim to synthesize a safer analogue of thalidomide. Recently, our group detected a higher frequency of alleles that impair the pro-angiogenic mechanisms of endothelial nitric oxide synthase (eNOS), coded by the *NOS3* gene. In this study we evaluated variable number tandem repeats (VNTR) functional polymorphism in intron 4 of *NOS3* in individuals with TE (38) and Brazilians without congenital anomalies (136). Haplotypes were estimated for this VNTR with previously analyzed polymorphisms, rs2070744 (−786C > T) and rs1799983 (894T > G), in promoter region and exon 7, respectively. Haplotypic distribution was different between the groups (p = 0.007). Alleles −786C (rs2070744) and 4b (VNTR), associated with decreased *NOS3* expression, presented in higher frequency in TE individuals (p = 0.018; OR = 2.57; IC = 1.2–5.8). This association was not identified with polymorphism 894T > G (p = 0.079), which influences eNOS enzymatic activity. These results suggest variants in *NOS3,* with pre-transcriptional effects as susceptibility factors, influencing the risk TE development. This finding generates insight for a new approach to research that pursues a safer analogue.

The consumption of thalidomide in early pregnancy was very common in the 1960s, as an antiemetic to control morning sickness^1^. The lack of awareness about the teratogenic properties of the drug led to the birth of ten thousand babies around the world affected by Thalidomide Embryopathy (TE). TE is a condition mainly characterized by limb defects^1^ which occur in 79–89% of the affected individuals^2^. Other organs frequently affected are the eyes, ears and heart^1^,^3^, occurring together with limb defects in around 19% of cases^2^. Severe internal organ defects were also diagnosed at the time, although the majority of the children affected by these anomalies died in the first years of life^3^. Nowadays, thalidomide is well known as a potent immunomodulator and antiangiogenic drug, properties that have led to its current use in treatment of many types of cancer (especially multiple myeloma) and immunological conditions^4^.

Many aspects of TE have been studied over the past few decades; however, its teratogenic molecular mechanism remains to be elucidated. One of the accepted hypotheses is that antiangiogenic property of thalidomide inhibits the proper development of new blood vessels, which are essential for limb development^2^,^5^.

The importance of nitric oxide in TE development has been reported in experimental studies assessing the teratogenic potential and antiangiogenic property of the drug^6^,^7^,^8^. Our group has recently published the first molecular study of TE in humans; we reported that the −786C (rs2070744) and 894T (rs1799983) alleles – which reduce the expression and activity of the *NOS3* gene, respectively, and their derived haplotype – were associated with TE, when compared with Brazilian people without congenital anomalies^9^.

In order to further explore and confirm the role that polymorphic variants in the *NOS3* gene result in susceptibility to TE, here we investigated the variable number tandem repeats (VNTR) of intron 4 (rs61722009) in the *NOS3* gene. Similarly to the previously investigated polymorphisms (−786T > C and 894G > T), the VNTR 4a/4b is common in different populations^10^. It has an important functional role in regulating eNOS and was believed to function either as an enhancer or repressor of *NOS3* gene transcription^11^,^12^, according to the number of repeats present in each allele^13^.

## Results

### Sample Characteristics

In total, our study involved 38 Brazilian individuals with TE, born between 1959 and 2010. The control group was comprised of 136 Brazilian individuals without congenital anomalies, born in similar regions and time periods as those TE individuals.

### Analysis of Allelic and Genotypic Frequencies

The distribution of all polymorphisms in both the sample groups was in accordance with the Hardy-Weinberg equilibrium.

Allele and genotype frequencies of the three polymorphisms are listed in Table 1. The frequency of alleles C and T in the polymorphism −786T > C was statistically different between the two sample groups (*p* = 0.022), corroborating results detailed in our previous study with a smaller sample size. However, the intron 4 VNTR and the 894G > T polymorphism was not significantly different between the two sample groups.

### Linkage Disequilibrium and Haplotype Analysis

Linkage disequilibrium analysis demonstrated that the three polymorphisms evaluated are in moderate disequilibrium (D′ > 0.3; p < 0.001).

Haplotypes and their frequencies in the two samples are described in Table 2. A comparison of the haplotype frequencies between groups revealed a statistically significant difference (*p* = 0.007).

### Evaluation of Risk Allele Association

Univariate logistic regression (Table 3) was performed to determine the association of *NOS3* with TE susceptibility. The alleles responsible for decrease in *NOS3* gene expression or eNOS enzyme activity were considered to indicate risk^8^. Based on this, we divided the sample in two groups: (i) individuals with at least one copy of the C, 4b, and T alleles of −786T > C polymorphism, the VNTR, and 894G > T polymorphism, respectively and (ii) individuals without such variants in at least one of the polymorphisms.

An association was not identified when all three polymorphisms were present together (*p* = 0.079). However, an analysis of the polymorphism in the promoter region (−786T > C) and the VNTR (performed in this study) showed that compared to the controls, case group showed a higher frequency of the alleles that reduce *NOS3* expression (Odds ratio (OR) = 2.57; 95% IC: 1,20–5,80; p = 0.018) (Table 3).

## Discussion

In this study, we have corroborated, using a larger sample size, that the −786C variant in the promoter region of the eNOS gene is more frequently present in individuals affected by TE than in those without congenital abnormalities. Moreover, we also identified an interaction between the −786T > C polymorphism and a functional intronic variant, which was previously not analyzed, as a genetic susceptibility factor to TE.

Nitric oxide is responsible for the regulation of vasodilation, exerting its effect through regulating the migration of endothelial cells^6^,^14^. It is also an important pro-angiogenic factor, stimulating the synthesis of cyclic guanosine monophosphate (cGMP) through soluble guanylyl-cyclase (sGC) activation^7^. Experimental models utilizing thalidomide and nitric oxide are well established in literature, and have helped to understand the physiological actions of nitric oxide in presence or absence of a highly anti-angiogenic teratogen. A previous study has demonstrated the relevance of nitric oxide in teratogenesis, especially during induction of limb reduction defects in mice models^15^. In addition, a role for nitric oxide in TE development has been suggested in thalidomide experimental models^6^,^7^,^8^. Thalidomide appears to attenuate nitric-oxide-induced migration of endothelial cells through suppression of cGMP levels, inhibiting angiogenesis at a cellular level through interaction with sGC^6^,^7^,^8^. It has not been determined whether a polymorphic eNOS protein would respond differently to the downstream effects of sGC-thalidomide interaction, and what consequences this polymorphic protein might have in terms of intensifying or decreasing the teratogenic potential of thalidomide. Nonetheless, it has been demonstrated that nitric oxide is able to rescue thalidomide induced teratogenicity in chicken and zebrafish embryos, reinforcing vasculogenesis and blocking apoptosis through modulating redox levels^8^.

Thalidomide is known as a multi-target drug with different therapeutic proprieties. It is not known whether these therapeutic proprieties overlap with its teratogenic ones. Anti-inflammatory actions of thalidomide occur mainly through the reduction of TNF-alpha mRNA levels^16^. TNF-alpha is a pro-inflammatory cytokine, stimulated by nuclear factor kappa-B (NF-KB), that able to reduce the half-life of eNOS mRNA^17^. NF-KB activates TNF-alpha to increase the expression of inducible oxide nitric synthase (iNOS) during inflammatory processes^18^. Thus, iNOS is principally responsible for high level of nitric oxide seen during inflammatory reactions, while eNOS has little activity in this context^19^. Overall, eNOS plays a fundamental role during limb outgrowth in the context of angiogenesis, while iNOS is irrelevant.

In regards to the effect of thalidomide upon oxidative stress, ROS levels are increased on drug exposure leading to a misbalance in the NF-KB pathway^20^. The consequences of this effect on NF-KB, its control of TNF-alpha, iNOS activation and eNOS repression remain unknown. Nonetheless, the high levels of ROS generated by thalidomide are also suggested to contribute to the drug’s teratogenic effect. Thus, it is possible that for carriers of alleles with reduced production of NO, the risks are not only for altered angiogenesis as well as for increased oxidative stress (due to thalidomide effect per se) causing synergistic effect susceptibility. Clearly these hypotheses should be further tested by *in vitro* and *in vivo* assays. As a complicating factor in this scenario, thalidomide is well known as an immunomodulatory drug, acting on interleukin (IL) and cytokines levels^20^. Hence, an individual’s response to thalidomide will also depend on their immunological status and developmental stage. Taking all this together, we believe that the role played by eNOS during thalidomide teratogenesis could be through multiple functions.

The fact that thalidomide is non-teratogenic in mouse and rats^21^, highlights the need of molecular studies in humans in order for us to fully understand the species-specific teratogenic properties of thalidomide. It has been reported that a human’s capacity of responding to an angiogenic stimulus is affected by genetic variants^22^, reflecting a variable susceptibility to develop a disease. The extent of thalidomide-induced teratogenesis is also dependent on genetic variability, and so must be considered in the context of TE.

**Figure 1:**
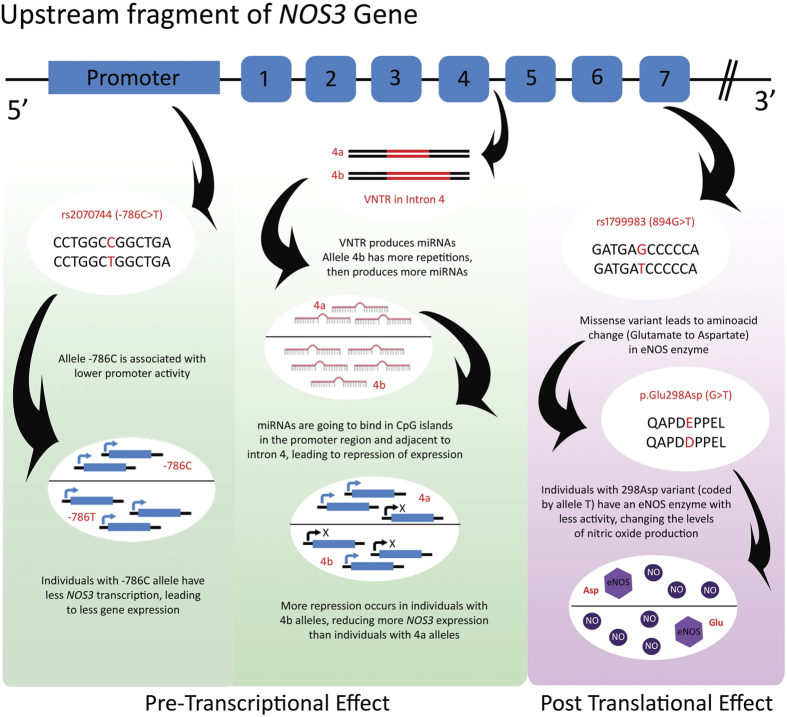
Pre-transcriptional and post translational effects of polymorphisms rs2070744, VNTR in intron 4 (rs61722009) and rs1799983.

As described in Fig. 1, experimental evidence suggests that the 894G > T polymorphism influences eNOS enzyme activity, altering production of nitric oxide^25^. On the other hand, the 786T > C polymorphism and the 4a/4b VNTR have been observed to regulate nitric oxide activity at the level of gene expression^24^,^26^. Absence of interactions between the 894G > T polymorphism and the two other polymorphisms in our univariate logistic regression model appears to indicate that thalidomide regulates *NOS3* at a pre-transcriptional level; this needs to be further investigated in studies evaluating additional gene regulation mechanisms. Regardless, the presence of alleles that result in reduced *NOS3* expression in thalidomide-affected individuals, as well as in other members of the population, results in a higher risk of developing the cited vascular conditions. The haplotype C/4b/T has previously been associated with lower levels of circulating nitric oxide^27^; many studies report C/4b/T as the susceptibility haplotype to conditions with impaired vascular function, such as hypertension^28^,^29^, preeclampsia^30^, and cardiomyopathies^31^.

Furthermore, the 27pb VNTR, at intron 4 of the *NOS3* gene, plays an important role in controlling *NOS3* gene expression. It is responsible for producing microRNA capable of altering DNA methylation and histone acetylation in the promoter region of the *NOS3* gene, and in regions adjacent to the VNTR itself^23^,^24^. It is believed that alleles with larger repeats, such as 4b, produce more microRNAs, therefore resulting in reduced *NOS3* gene expression compared to 4a^13^. The polymorphism 894G > T has been previously associated with congenital heart defects (CHD), especially when together with tobacco exposure in pregnancy^33^. Recent research has evaluated this polymorphism, with −786C > T in a Chinese sample, although an association was not identified^34^. In our study, it was not possible to determine an association between NOS3 risk haplotypes and congenital anomalies (data not showed), possibly due to the evaluated sample size”.

It was reported in the 1960’s that TE occurs in 10–50% of all babies exposed to thalidomide *in utero*^32^. Genetic variability may help to explain why some individuals are apparently resistant to the drug’s teratogenic effects while others are more susceptible. The best approach in a case-control study would be to access people who were exposed to thalidomide, although did not develop TE, as a control group. Unfortunately, such data does not exist since the thalidomide tragedy occurred more than fifty years ago and unaffected individuals were not identified at the time. The control group in the current study is composed of individuals born at the same time as individuals affected with TE and in the same Brazilian regions. In these regions, thalidomide was highly available and could be bought without prescription. However, it is not possible to confirm if any individuals in the control group were exposed to the drug. Clearly, not only this variable was lost, but many other variables needed to evaluate susceptibility or phenotypes characteristic of individuals affected by TE.

In the present study we suggest that individuals with alleles −786C and (VNTR) 4b may be more sensitive to thalidomide than those without these variants. This may help to explain why some individuals were exposed to thalidomide but did not develop TE. Further analysis with a larger sample size should help evaluate endophenotypes of TE and vascular conditions in both groups. Studies focusing on thalidomide-induced susceptibility to embryopathy in angiogenesis (as well as in other pathways) might help elucidate the molecular mechanisms underlying thalidomide teratogenesis. This may contribute to our understanding and treatment of diseases in which these alleles are also considered risk factors. The alleles investigated have high frequency across different populations (−786C varying from 12–44%, and VNTR 4a with a 12–20% frequency)^10^. Hence, it is also relevant to look at presented results with focus and view on personalized medicine strategies. Pharmacogenetic studies, especially in Erithema Nodosum Leprosum and Multiple Myeloma (the main conditions treated by thalidomide and its analogues), can help not only to identify genetic profiles with a better response to the drug, but also to look for alternative drugs when the therapeutic benefits of thalidomide are not reached for these people. Further, *in vitro* and *in vivo* research is essential to understand the role of the *NOS3* polymorphisms analyzed in thalidomide teratogenesis as well as in therapeutics settings and pharmacogenetics approaches. We believe this is a first step that can put forward different therapeutic strategies involving thalidomide.

## Materials and Methods

### Ethical Aspects

This study was approved by the Research Ethics Committee of the Clinical Hospital of Porto Alegre (number 10-0244). The methods were carried out in accordance with the approved guidelines.

### Recruitment and Sample Collection

Individuals with TE were recruited through the Brazilian Association of People with Thalidomide Syndrome; all subjects signed the informed consent. The congenital anomalies in these individuals were assessed through the TE diagnostic guideline previously published^9^. Only individuals displaying phenotypes compatible with TE were included in this study. Twenty-eight individuals completed a clinical questionnaire that characterized their congenital anomalies according to organs and systems affected^35^.

Saliva collection was performed through Oragene-DNA OG-500 (DNA Genotek^®^) and DNA was obtained according to the manufacturer’s instructions.

The control group consisted of Brazilians without any congenital anomalies. DNA samples from the control subjects were stored in the Genetics Department of our institution; they have already been used in previous epidemiological studies^36^,^37^. Gender, age, date, and place of birth information were also collected from subjects.

### SNPs and VNTR Genotyping

The 27bp VNTR of intron 4 (rs61722009) was genotyped according to Marroni^38^. A small percentage (10%) of the sample was selected randomly to confirm the result by Sanger sequencing in equipment ABI 3730XL (Applied Biosystems^®^).

The polymorphisms rs2070744 and rs1799983 (−786T > C and 894G > T, respectively) were also genotyped in the new cases and controls; these samples were genotyped by TaqMan^®^ Genotyping Assay (Applied Biosystems) as previously described^39^.

### Evaluation of Linkage Disequilibrium and Haplotypes

Linkage disequilibrium between variants was estimated using the MLocus program^40^, and the haplotypes were derived using the PHASE v.2.1 software (University of Chicago, Chicago, IL, USA).

### Statistical Analysis

All statistical analyses were evaluated in SPSS^®^ program, version 20 (SPSS, IBM, USA). All tests were two-tailed and the Bonferroni correction was applied in all sample groups.

## Additional Information

**How to cite this article**: Kowalski, T. W. *et al.* New Findings in eNOS gene and Thalidomide Embryopathy Suggest pre-transcriptional effect variants as susceptibility factors. *Sci. Rep.* **6**, 23404; doi: 10.1038/srep23404 (2016).

## Figures and Tables

**Table 1:** Allelic and genotypic frequencies of the polymorphisms of the *NOS3* gene in individuals with thalidomide embryopathy, as well as in Brazilian individuals without congenital anomalies (unaffected group).

Gene	Polymorphism	Genotype/Allele	Affected		Unaffected		P-Value^a^
			N	%	N	%	
*NOS3*	rs2070744	CC	10	26.3	17	12.5	0.060
	(C/T)	CT	17	44.7	57	41.9	
		TT	11	28.9	62	45.6	
		C	37	48.7	91	33.5	0.022
		T	39	51.3	181	66.5	
	rs61722009	4b4b	27	71.1	81	61.8	0.263
	(VNTR)	4b4a	11	28.9	41	31.3	
		4a4a	0	0.0	9	6.9	
		4b	59	85.5	203	77.5	0.149
		4a	11	14.5	59	22.5	
	rs1799983	TT	5	13.2	13	9.6	0.360
	(T/G)	TG	21	55.3	63	46.7	
		GG	12	31.6	59	43.7	
		T	31	40.8	89	33.0	0.221
		G	45	59.2	181	67.0	

^a^Chi-Square Test.

**Table 2:** The inferred haplotypes and haplotypic frequencies in individuals with thalidomide embryopathy, as well as in Brazilian individuals without congenital anomalies (unaffected group).

Gene	Haplotype	Affected		Unaffected		P-Value^a^
		n	%	n	%	
*NOS3*	T 4b G	24	31.6	120	44.1	0.007
	T 4b T	9	11.8	30	11.0	
	T 4a G	6	7.9	30	11.0	
	T 4a T	0	0.0	1	0.4	
	C 4b G	10	13.2	6	2.2	
	C 4b T	22	28.9	56	20.6	
	C 4a G	5	6.6	26	9.6	
	C 4a T	0	0.0	3	1.1	

^a^Chi-Square Test.

**Table 3:** Univariate logistic regression model to assess risk alleles in individuals with thalidomide embryopathy and in individuals of the unaffected group.

Risk Alleles of *NOS3* Gene	Presence		Absence		Odds Ratio (95% IC)	P-Value
	n	%	n	%		
(−786)C + (VNTR)4b	91	53.8	78	46.2	2.570 (1,20–5,80)	0.018
(−786)C + (VNTR)4b + (894)T	68	52.6	101	36.6	1.921 (0,93–4,01)	0.079

CI: confidence interval; Polymorphisms used in the model are rs2070744 (−786C), rs61722009 (VNTR), and rs1799983 (894T).
